# Stereotactic Body Radiation Therapy (SBRT) as Salvage Therapy for Oligorecurrent Pleural Mesothelioma After Multi-Modality Therapy

**DOI:** 10.3389/fonc.2019.00961

**Published:** 2019-09-26

**Authors:** Christina Schröder, Isabelle Opitz, Matthias Guckenberger, Rolf Stahel, Walter Weder, Robert Förster, Nicolaus Andratschke, Olivia Lauk

**Affiliations:** ^1^Department of Radiation Oncology, University Hospital Zurich, Zurich, Switzerland; ^2^Department of Thoracic Surgery, University Hospital Zurich, Zurich, Switzerland; ^3^Department of Medical Oncology, University Hospital Zurich, Zurich, Switzerland

**Keywords:** SBRT, malignant pleural mesothelioma, local recurrence, oligoprogression, retrospective analysis

## Abstract

**Introduction:** Therapy options for patients with oligoprogressive malignant pleural mesothelioma (MPM) are limited. Stereotactic Body Radiotherapy (SBRT) may be a promising therapeutic option, as it delivers a localized ablative dose of radiation and therefore balances efficacy and treatment related toxicities. The intent of this retrospective analysis was to evaluate the feasibility of SBRT for limited pleural recurrences.

**Methods and Materials:** This retrospective single-institution study is based on the 21 consecutive patients treated with hypofractionated radiotherapy for oligoprogressive MPM. Clinical and radiological data was collected at regular follow-up visits including toxicity, local control and survival.

**Results:** At primary diagnosis, 57% of the patients presented with stage III disease. Initial treatment of MPM consisted of induction chemotherapy (*n* = 12) prior to a macroscopic complete resection (*n* = 18). Three patients received additional intracavitary chemotherapy and another three patients were treated with chemotherapy alone without another treatment at the time of first diagnosis. A total of 50 lesions in recurrent MPM were treated with SBRT. The median number of radiotherapy fractions was 5 (range 3–20) with a median dose per fraction of 5 Gy (range 2.5–12.5 Gy). The median total treatment dose was 30 Gy (20–50 Gy) with a median prescription isodose line (IDL) of 65% (65–100%). Median follow-up of all patients from diagnosis was 28 months (range 7–152 months). Analyzing all lesions separately, the 12-months-local control from SBRT was 73.5%. The median progression free survival (PFS) after SBRT was 6 months (range 0–21 months) and the median OS from first first SBRT was 29 months (range 0–61 months). Only one patients experienced above Grade 3 toxicities.

**Conclusion:** This analysis demonstrates the feasibility of a SBRT approach for oligorecurrent MPM. SBRT was well-tolerated even after multiple repetitions and local control was high with a promising median OS.

## Introduction

Malignant pleural mesothelioma (MPM) still has a devastating prognosis. Even after recent advances in therapy options within a multi-modality therapy setting combining chemo- and/or radiotherapy to mesothelioma resection, the median survival is up to 23 months (1–5).

The main limiting factor until today is a high local recurrence pattern for this disease. Due to anatomical restraints, microscopic tumor burden will be eventually left behind even after radical surgery. The role of adjuvant radiotherapy in this setting remains unclear. Although *in-vivo*, MPM cell lines shown a great variety of radiosensitivity, including highly radiosensitive lines, data regarding clinical outcome remains inconclusive (6–9).

In case of recurrent disease after initial multimodal treatment, standardized treatment recommendations for effective salvage strategies treatment are needed. According to ASCO guidelines, radiation therapy may be offered to patients with localized asymptomatic recurrence (moderate strength of recommendation). The dosage of fractionated radiotherapy depends on the site and extent of disease and should be discussed on an individual basis (10). Especially patients with very limited local pleural recurrences represent a challenge, as the optimal treatment strategy balancing toxicity and efficacy has not been defined yet (11). These patients may be candidates for a local ablative treatment and may benefit from an extended progression free survival (PFS) until systemic therapy is indicated. Stereotactic body radiotherapy (SBRT) might be a promising option for these patients since it delivers a local ablative biologic dose of radiation and was recently explored in the oligoprogressive setting of solid tumors with excellent local control rates, encouraging outcome, and low severe toxicity (12, 13). The benefit of locally ablative therapy could be shown for other tumor entities, e.g., by Gomez et al. in patients with stage IV non small cell lung cancer (NSCLC) with up to three metastases or Palma et al. in patients with up to five metastases of different primary tumors. Both could show a statistically significant improvement in overall survival by the use of SBRT, e.g., 41.2 months vs. 17 months for Gomez et al. (14, 15).

The intent of this retrospective analysis was to evaluate the feasibility of a novel strategy to integrate SBRT as first salvage therapy for limited pleural recurrences in pleural mesothelioma.

## Methods and Materials

### Patient Characteristics

Our instituitonal database lists patients since 1999 and is simultaneously of prospective nature.

Between 2010 and 2018, 21 patients with the histo-pathological diagnosis of MPM were treated with hypofractionated radiotherapy for thoracic oligometastatic progression.

Most patients initially presented with IMIG stage III disease (57%). Only one patient had distant metastases upon first diagnosis. The median age at first diagnosis was 65 years with a range from 33 to 75 years.

For initial treatment of MPM, 18 patients (ECOG 0-1) had received a macroscopic complete resection (MCR), of which 12 patients received systemic induction (platinum based) therapy prior to the surgical resection. Three patients were treated with chemotherapy alone without any other treatment. MCR consisted of (extrapleural) pleurectomy/decortication and one pleurectomy. In some cases additional intracavitary cisplatin/fibrin application was performed within our clinical phase I/II trial (Intracavitary Cisplatin-Fibrin Localized Chemotherapy after Pleurectomy/Decortication for the Treatment of Patients with Malignant Pleural Mesothelioma (INFLuenCe-Meso I/II). Further patient characteristics are shown in Table 1.

**Table 1:** Patient characteristics.

	***n***	**%**
**Sex**		
Male	17	81
Female	4	19
**Histology**		
Epithelioid	17	81
Sarcomatoid	2	9.5
Biphasic	2	9.5
**Imig stage**		
I	3	14.3
II	4	19
III	12	57.1
IV	2	9.5
**Pre-SBRT therapy modalities**		
Induction systemic therapy	12	67
Surgical resection	18	86
Intracavitary chemotherapy	3	25
Systemic therapy alone	3	14
Total	21	100

### Radiation Treatment Planning and Delivery

Planning CT was acquired as 4D-CT with retrospective amplitude-based image sorting. In addition, a 3D-CT was performed in free breathing to allow for contrast i.v. injection.

Gross tumor volume (GTV) was contoured as the visible tumor in the planning CT supplemented by information from i.v. contrast 3D-CT or further imaging including FDG-PET or magnetic resonance imaging (MRI) if available. In FDG-PET CT scans, the FDG active lesions with an visible correlate in the i.v. CT scans were contoured. No additional clinical target volume (CTV) margin was added (i.e., GTV = CTV).

The internal target volume (ITV) was generated as a composite GTV from the different amplitude-based reconstructed CT scans complemented by a margin of 5 mm to derive the planning target volume (PTV). Treatment planning and delivery was done with either conformal or intensity-modulated (VMAT) techniques.

All plans were calculated by a radiation therapy technologist using common constraints for the organs at risk and target prescription standards and were multidisciplinary reviewed. For treatment planning, Eclipse software™ (Varian medical systems) was used. Patients were treated with either 6 or 18 MV. If necessary immobilization by individualized vacuum cast or abdominal compression was used.

### Endpoints and Toxicity Definitions

During treatment, all patients were monitored daily for acute treatment related toxicity. Follow-up 6 weeks after completion of SBRT and every 3–4 months thereafter included physical examination and CT, PET-CT and/or MRI scans until tumor progression. Toxicity was scored according to the National Cancer Institute for Common Terminology Criteria for Adverse Events (CTCAE) v5.0 criteria. Local failure of a metastatic lesion was defined as either reappearance after complete remission or re-growth after initial partial response on follow-up CT or MRI scans.

### Statistical Analysis

Overall survival and PFS were calculated according to the Kaplan-Meier method. Overall survival (OS) was calculated from first diagnosis until death or last follow-up, PFS from SBRT until tumor relapse or last follow-up. Regarding radiation treatment parameters, descriptive statistics were calculated. Additionally, the biological effective dose (BED) as well as the 2-Gy equivalent dose (EQD2) were calculated according to the linear-quadratic (LQ) formalism using an alpha-beta for tumor tissue of 10 Gy. For statistical analysis, SPSS version 25 was used.

## Results

### Radiation Therapy

A total of 21 patients received 1 course of radiation treatment, 10 of those received a second and 4 a third course of RT. A total of 50 lesions in recurrent MPM were treated with SBRT. The median number of PTVs treated during a course was 1 (range 1–3). 75 to 100% were treated with a locally ablative dose in an oligorecurrent setting at all courses, but up to 25% of patients were also treated with a palliative analgetic approach. Two patients received concurrent systemic therapy (Pembrolizumab). The median time between diagnosis of MPM and first radiation treatment was 15 months (range 5–90 months).

The median number of fractions at all courses was 5 (range 3–20) with a median prescription dose per fraction of 5 Gy (range 2.5–12.5 Gy). The median total prescription dose was 30 Gy (20–50 Gy) with a median prescription isodose line (IDL) of 65% (65–100%).

The median PTV volume of all lesions was 40.1 cc (range 3.3–774.3 cc). The median EQD2 (2-Gy-eqivalent dose) was 44.87 Gy (range 23.49–88.34 Gy). A detailed overview of the radiotherapy treatment parameters separately by RT courses are shown in Table 2. If there were multiple lesions treated at one timepoint the sum plan of all lesions was used if they were in relevant proximity.

**Table 2:** Dosimetric parameters of RT courses.

**RT course**	**1st**		**2nd**		**3rd**	
**PTV**	**Median**	**Range**	**Median**	**Range**	**Median**	**Range**
Volume (cc)	47.8	3.3–754.2	29.8	4.9–264.00	432.65	38.60–774.30
Dose/fraction mean (Gy)	6.6	2.5–14.45	6.3	2.55–13.87	3	2.61–9.14
Total dose mean (Gy)	38.22	20.11–50.00	32.32	30.00–57.50	33.69	30.00–45.70
BED mean (Gy)	56.82	28.19–106.00	51.35	38.45–99.37	43.19	39.00–87.48
EQD2 mean (Gy_10_)	47.35	23.49–88.34	42.79	32.04–82.81	35.99	32.5–72.90

### Clinical Outcome

Median follow-up of all patients from diagnosis on was 28 months (range 7–152 months). At the time of analysis, 11 patients were still alive.

#### Local Control and Pattern of Progression

Intrathoracic out-of-field or in-field recurrence after SBRT was observed in up to 62% of patients. After the first course of SBRT, a total of 13/21 patients had a thoracic recurrence (11 out-of-field, 2 in-field). After the second and third course the number of patients with thoracic recurrence was 6/10 and 2/4, respectively (each with 50% in-field-recurrence).

**Figure 1:**
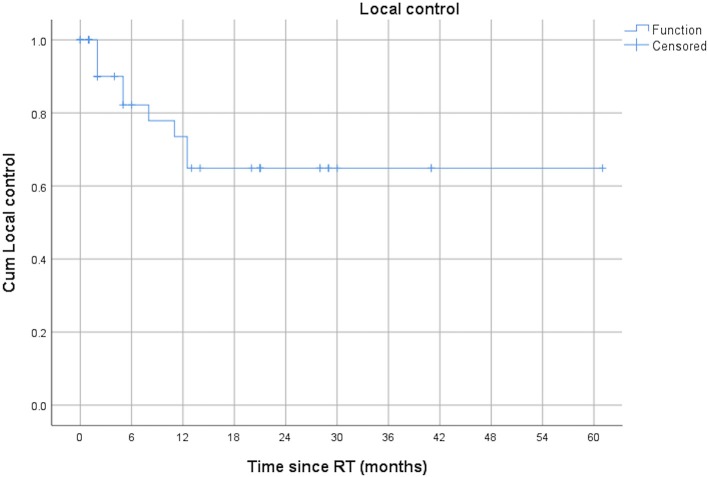
Local control for all lesions after repeated courses of SBRT.

**Figure 2:**
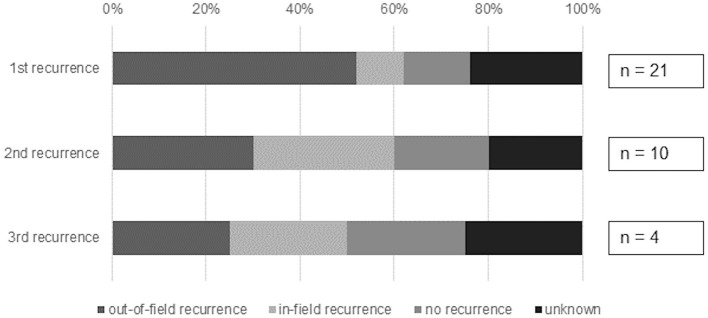
Patterns of failure after repeated courses of SBRT for oligometastatic recurrence.

When looking at all lesions separately the 12-months-local control of the irradiated lesions was 73.5%. Figure 1 shows the local control after repeated courses of SBRT and Figure 2 the patterns of failure.

Patients with a recurrence after the first SBRT received a variety of treatment modalities. Most patients received another SBRT (54%) and /or systemic therapy (38%). One patient received a pleurectomy/decortication. In case of another recurrence, most patients were treated with systemic therapy (86–100%).

#### PFS and OS

**Figure 3:**
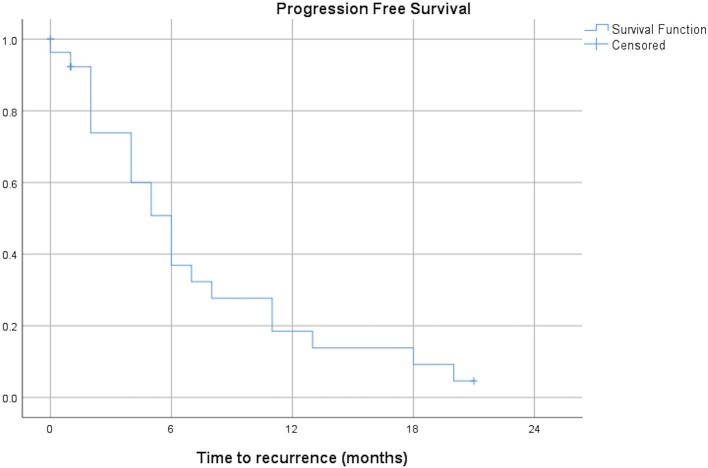
Progression free survival after first SBRT (months, *n* = 21).

**Figure 4:**
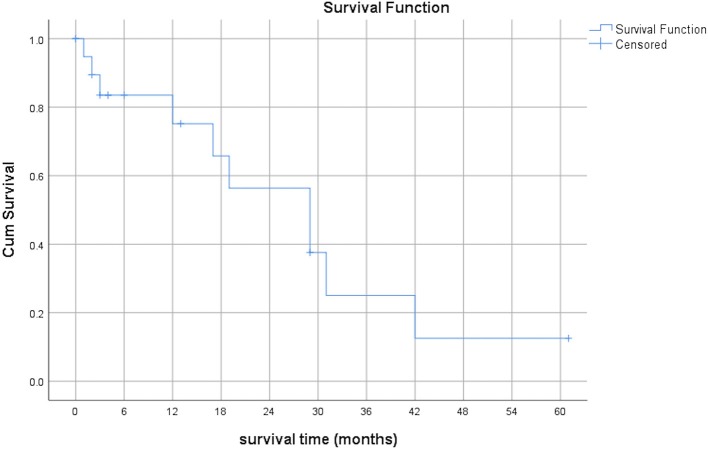
Overall survival after first SBRT (months, *n* = 21).

The median PFS after first SBRT was 6 months (range 0–21 months) and the median OS after first SBRT was 29 months (range 0–61 months). The OS at 3 years was 9.5%. Figures 3, 4 show the PFS and the OS from first SBRT.

#### Toxicity

Overall, the radiation treatment was very well-tolerated. Only 1 patient experienced ≥ Grade 3 toxicity. This patient whose MPM was infiltrating the esophagus and who received RT of the esophagus and mediastinal lesions was hospitalized during treatment due to upper gastrointestinal bleeding. The same patient died 3 months later due to massive esophageal bleeding and progressive disease.

## Discussion

In this retrospective single institution study, we analyzed the feasibility of SBRT as a salvage procedure in 21 patients with oligoprogressive MPM. Therapy options for patients with oligoprogressive malignant pleural mesothelioma (MPM) are limited. However, there is few data about SBRT as a salvage strategy for oligoprogressive MPM. Oligoprogression, wheter in MPM or otherwise, is not consistently defined throughout the literature. For our analysis, we specified it as a maximum of three lesions. SBRT is a promising treatment option in this setting as it delivers a local ablative radiation dose as recently explored in an oligometastatic setting of solid tumors with excellent local control rates, encouraging outcome, and low severe toxicity (14–18).

In our cohort, patients were treated with a median EQD2 of 44.87 Gy_10_. Local control was very promising with a 12-months local control of the irradiated lesions of 73.5%. The applied radiation doses were not in the range of the ablative doses applied in phase I or II studies regarding oligometastatic lesions of other primary disease. In the NRG-BR001 and SABR-COMET trials, the treatment dose was 30–50 Gy in 3–5 fractions and 30–60 Gy in 3–8 fractions, respectively (15, 17). Nevertheless, the local control in our patient cohort appears to be very promising. Regarding SBRT in MPM patients there is only one case report about a patient receiving Cyberknife treatment (70 Gy/5 fx) for a focal paravertebral recurrence after MCR in MPM who remained disease-free at 40 months (19). Additionally, two case series exist about patients receiving palliative stereotactic IMRT by Munter et al., but not for an oligoprogressive setting as in our cohort (20, 21).

Looking at efficacy, although our results are promising, there are certainly challenges regarding the definition for an optimal target volume. Even with PET-CT based planning, lesions might be missed or underestimated due to the disease‘s nature, respectively its infiltrating pattern. Hence, not all of the lesions might be covered with a sufficient dose, resulting in recurrences. The close neighborhood to abdominal organs at risk (OARs), especially if targeting lesions close to the diaphragm, might also lead to dose compromises in the target volume necessary for sparing the OAR.

When looking at safety, our toxicity profile with only 1 patient experiencing ≥ grade 3 toxicity due to esophagus infiltration is promising. The patient was hospitalized during treatment due to resulting upper gastrointestinal hemorrhage and eventually died of a massive bleeding later on. We interpreted this as a combination of the local radiation therapy in addition to the infiltrating and progressive disease. There were no signs of any severe pneumonitis in our cohort.

Due to the high local control, promising OS and low toxicity profile we propose that SBRT may be a promising approach to provide effective local control in a short overall treatment time to delay systemic therapy until further progression in selected patients.

We are aware of the limitation of this case series concerning the inhomogeneity of the patient cohort with their different therapies applied beforehand and divergent time points of radiotherapy. Nevertheless, oligoprogression is observed in MPM and SBRT appears to be feasible and safe, especially for patients with a reduced general condition after multiple previous therapies. Further studies are needed to determine the role of SBRT and should focus on optimizing fraction schemes as well as exploring the influence on the patient's quality of life.

## Data Availability Statement

The dataset for this manuscript are available upon request. Requests to access the datasets should be directed to the corresponding data.

## Ethics Statement

The local ethics committee approved this retrospective analysis of the mesothelioma data base (StV 29-2009, EK-ZH 2012-0094).

## Author Contributions

CS and OL were responsible for conception and design, collection, and analysis of data as well as manuscript writing. NA was responsible for conception/design, collection and analysis of data, administrative support, and provision of patients. IO, MG, WW, RF, and RS provided administrative support and patients.

### Conflict of Interest

The authors declare that the research was conducted in the absence of any commercial or financial relationships that could be construed as a potential conflict of interest.
